# Automobile Licensure

**Published:** 1915-06

**Authors:** 


### Automobile Licensure.

As a matter of courtesy, on account of the meeting of the Medical Society of the State of New York in Buffalo, Canadian physicians were allowed to enter Buffalo by way of Fort Erie, with automobiles, without securing N. Y. State licenses. This privilege was granted for Niagara Falls, but not Tonawanda, so that the selection of route was limited.

This action suggests some general remarks as to automobile licensure, in which the medical profession is interested, not only as one of public policy but because no class of men in the country uses automobiles to a greater proportionate degree than physicians, with the sole exception of presidents. Article 4, Section 1, of the U. S. Constitution provides that "full faith and credit shall be given in each state to the public acts, records and judicial proceedings of every other state" and congress is empowered to prescribe the construction of such acts. Section 2 states that the "citizens of each state shall be entitled to all privileges and immunities of citizens in the several states." Article 1 gives congress authority over foreign and interstate commerce and over post roads.

It is inevitable that a person, going from his own to another state, should receive certain benefits of a general nature, for which the citizens of that state pay and for which he himself pays only as he purchases lodging, food and other necessities and luxuries and thus contributes indirectly to the prosperity of the state in which he is a guest. In the long run, this immunity of the guest from direct taxation, is automatically balanced. Up to the development of special taxation of automobiles, we do not recall any precedent for the taxation of interstate guests nor any abridgement of the period of their sojourn in another state. In particular, we do not recall any precedent for the imposition of a state tax on any vehicle or means of transportation, coming from another state. The special taxation and licensure of an automobile in the state of residence has even been claimed to be unconstitutional or, at least, contrary to the spirit of the law but is usually conceded to be proper, partly to control a possible source of danger, partly because of excessive destruction of highways, partly as a protection to the owner in case of theft, partly as a means of raising funds largely, though not exclusively, employed to the advantage of the automobilist.

As all of these purposes of taxation and licensure are served by their application in a single state of residence, and in view of the precedent against taxation of a non-resident—excepting, of course for local property—it would seem that the constitutional provisions cited amply support the contention that taxation of an automobile in one state, that of actual residence, should afford immunity from further taxation throughout the United States. This problem applies with especial force to persons whose homes are near the boundaries of two or more states and, with the most hardship to residents of the District of Columbia. We do not care to consider the complicated problem of national licensure, further than to admit certain practical advantages and to say that we have not the legal acumen to appreciate why it is any more unconstitutional in the invasion of states' rights than the local application of the Harrison law. The practical point is that one, single, license, should apply to the operation of an automobile at will throughout the entire United States, without a time-limit, any more than in the case of a traveler by train or boat, or buggy, or on foot. If this principle could be established, the securing of international reciprocity, especially with Canada, would be a simple matter.

It scarcely requires argument that such an arrangement would, in the long run, conduce to the prosperity of the whole country. States with a large automobile "population" and with an almost necessary consequence of an abundance of good roads, would, indirectly, receive benefits far greater than can be derived from direct double taxation. States not thus equipped would receive benefit somewhat in proportion to their deserts and would be stimulated to improve their roads and thus to increase the results both from direct taxation of residents and indirect receipts from visitors.

If this contention is not correct, let us be consistent and tax every other vehicle, horse-drawn carriage, locomotive or passenger car, entering a state from another state, place a time limit on the period which a person may spend outside his own state, without a special tax, in short, establish interstate custom houses.

